# Effects of Sitka spruce masting on phenology and demography of siskins *Spinus spinus*

**DOI:** 10.1038/s41598-021-84471-8

**Published:** 2021-03-01

**Authors:** Euan N. Furness, Robert W. Furness

**Affiliations:** 1 Science and Solutions for a Changing Planet DTP and Department of Earth Sciences and Engineering, Imperial College London, South Kensington Campus, London, SW7 2AZ UK; 2 Institute of Biodiversity, Animal Health and Comparative Medicine, University of Glasgow, Glasgow, G12 8QQ UK

**Keywords:** Animal behaviour, Behavioural ecology, Boreal ecology, Conservation biology, Stable isotope analysis

## Abstract

Masting behaviour of Sitka spruce *Picea sitchensis* may influence Eurasian siskin *Spinus spinus* breeding ecology as breeding siskins specialize on spruce seeds. We caught siskins and other small passerines over 16 years using mist nets adjacent to large plantations of mature Sitka spruce. We sexed, aged, measured and weighed the birds and collected feather samples from fledglings to measure nitrogen and carbon stable isotope ratios. Siskins departed in late summer, and returned, and bred earlier in years of higher cone abundance. Nitrogen and carbon isotopes indicated that siskins fed their chicks on Sitka spruce seeds in most years, and more so in years of high cone production. More siskins were caught following heavy rainfall, when the cones had closed, encouraging the birds to seek alternative food sources. Fledglings were not heavier or larger in years with higher cone crops but were more numerous. However, the age ratio of siskins caught the following year was unaffected by cone crop. Given their reliance on Sitka spruce seeds, climate change may have a major impact on siskin numbers by altering the availability of Sitka spruce seeds, either through changes in masting patterns or cone opening, or due to climate-related changes in forestry practices. Siskins represent a valuable study system to conservation ecology, where a native species is ecologically reliant on introduced taxa.

## Introduction

The Eurasian siskin *Spinus spinus* is a widespread breeding species in conifer forests in Europe and east-Asia, and is common in mature Sitka spruce *Picea sitchensis* plantations in the west and north of the British Isles^1^. Siskins and crossbills Loxia sp. specialise in feeding on seeds from conifer cones while the cones are on the tree: however, although crossbills can open closed cones^2^, siskins can only extract seeds from already-open cones^3^. The breeding range and numbers of siskins have increased dramatically in Britain following the increase in plantation forestry and the introduction of Sitka spruce to large areas of upland Britain^4^, with up to 3.5 million breeding pairs in Scotland in the 2000s, almost all in mature conifer plantations^3^. Since Sitka spruce is not native to Britain, this represents a study system where a once-scarce native animal has been able to benefit greatly from the introduction of a non-native food supply. Potential impacts on the wider bird community of the increase in abundance of siskins have not been explored. Young in the nest are fed on conifer seeds and invertebrates^5^, and Newton (1972)^6^ suggested that first broods may be fed more on spruce seeds, and second broods more on pine seeds and invertebrates. However, the relative importance of Sitka spruce seed in the diet of siskin chicks remains uncertain. Sitka spruce cones ripen in September–October and seeds can be shed during dry weather from then onwards^7–10^. Summers (2018)^10^ found that Sitka spruce cones in Speyside, Scotland, retained most of their seeds through the winter due to the damp conditions, and then shed seed through the following spring and summer, with about 10% of seeds still in the cones in July. This suggests that breeding siskins would be able to access some Sitka spruce seeds throughout their breeding season (March to July). Siskins tend to leave their breeding areas in Scotland in late summer, possibly in response to the spruce cones being emptied of seed, and return in early spring, by which time a new cone crop is available. Ringing data show movements from Scottish breeding areas to England and, in some cases, to southern Europe^11^. Before returning to their breeding areas, siskins are late-winter visitors to many gardens throughout Britain and Ireland, especially in years when the Sitka spruce cone crop is poor^12^. Siskins also arrive in Britain in autumn from Scandinavia, but the relative numbers of migrants and local birds are unclear^3^. Reviewing the seasonal movements of siskins based on bird-ringing data in Britain and Ireland, Martin (2002)^11^ stated that ‘The siskin remains a relatively difficult species to study during the breeding and post-breeding dispersal periods. Increased ringing activity at breeding sites, and studies that improve our understanding of the factors that drive variability from year to year should be encouraged in order to provide a more balanced understanding’.

Sitka spruce shows masting seed production, with years of high cone production synchronous throughout Britain, interspersed with years of low cone production^13^. Masting is thought to be controlled by climate, and is thus susceptible to climate change^14–17^. Siskins start to breed earlier in years with high production of Sitka spruce cones^18^. It has also been suggested that weather conditions influence feeding because cone scales close during damp weather, making conifer seed less accessible and therefore forcing siskins to find other food supplies^3^. No studies have considered whether in years of poor cone crop, siskins, which are very abundant in areas with extensive plantation forestry, compete more with other bird species for alternative food supplies.

In this study, we investigated the relationships among siskins, Sitka spruce cone crop and rainfall based on approximately weekly mist net catches of siskins and other small passerines from 2005 to 2020 at a site in the west of Scotland, adjacent to a large area of mostly mature plantation forestry. We gathered data on numbers of birds caught per day, ages, weights, wing lengths, and brood patches. Numbers caught reflect movement of birds into gardens to feed, and so are influenced both by numbers present and by use of a variety of potential alternative food supplies, which are also used by a range of other small passerine birds. We collected Sitka spruce seeds, and feathers from fledgling siskins and other birds, for stable isotope analysis of nitrogen and carbon, to investigate variations in diet in relation to cone crop. Passerine chicks grow their first set of feathers before leaving the nest, and the isotopic composition of these feathers in fledglings thus reflects the food provided by their parents while the chicks are in the nest. The nitrogen isotope ratio shows strong and consistent enrichment of the heavier isotope with each increase in trophic level^19^, and we therefore hypothesized that the nitrogen isotope data would show that siskins fed their nestlings at a lower trophic level than other small passerines in the area (i.e. more on Sitka spruce seeds and less on invertebrates), and at a lower trophic level in years with larger Sitka spruce cone crops. The carbon isotope ratio varies considerably among plant species^20^, and we therefore also expected to detect a difference in carbon isotope ratios between siskins and other small passerines, reflecting a strong dependence of siskins on Sitka spruce seed to feed their nestlings. However, in contrast to the nitrogen isotope ratio, we were unable to predict exactly how the carbon isotope ratio would differ among bird species at the study site, given that it is influenced by the specific ecology of producers in ecosystems^21,22^, with a relatively small effect of trophic level^19,23^. Nevertheless, we hypothesized that we would observe consistent changes in carbon isotope ratio in fledgling siskins between years of high and low cone crop, and differences between siskins and other small passerines, owing to differences in diet.

Our aim was to obtain a better understanding of how masting behaviour of Sitka spruce influences siskin breeding phenology and ecology, and to explore the implications that this influence has for the conservation of siskins, and for conservation of species in analogous positions worldwide. We addressed this aim by investigating the following hypotheses:

- Siskins arrive in the breeding area earlier when the Sitka spruce cone crop is larger;
- Siskins breed earlier when the Sitka spruce cone crop is larger;
- Siskins chicks are fed at a lower trophic level than chicks of other small passerines in the area;
- Siskins visit and feed in gardens in larger numbers in years with lower Sitka spruce cone crops;
- Siskins are more likely to be caught in mist nets immediately following periods of higher rainfall (presumably as a result of their being more likely to attend feeders in gardens);
- Siskin fledglings are more abundant, and of higher body quality, in years with higher Sitka spruce cone crops;
- The siskin age ratio, based on mist net catches, reflects the cone crop in the previous year.

## Results

### Timing of breeding start

**Figure 1:**
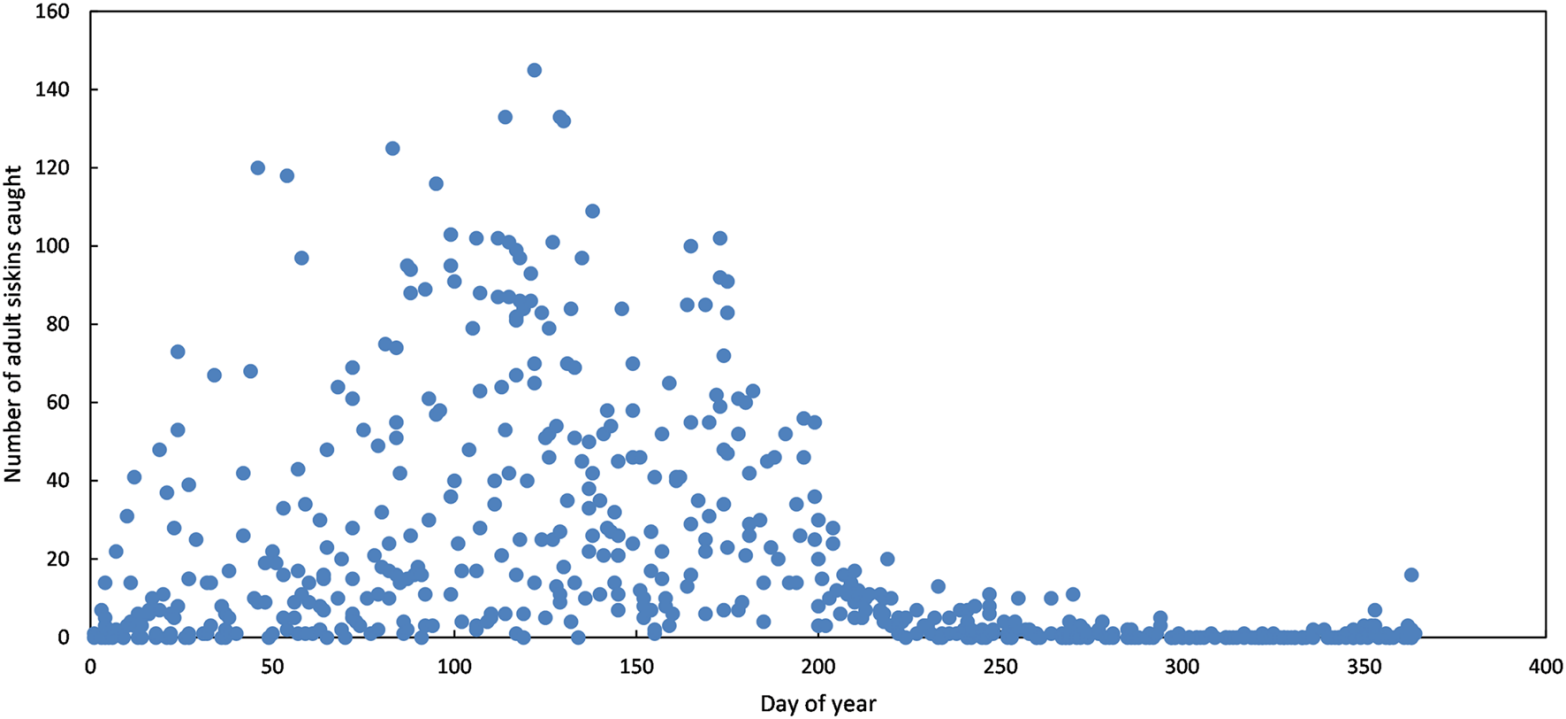
Seasonal variation in the number of adult siskins caught per day. Each point represents one catching session between 2005 and 2020. Day of year starts on the 1st of January.

Numbers of adult siskins (Euring age codes 4, 5 or 6) in daily catches across all years (2005–2020 combined) varied considerably, from zero to > 140, but showed a clear seasonal pattern. A median of zero adult siskins were caught between day 280 (7 October) and day 349 (15 December) for all 81 catches during that period (Fig. 1), and on average, numbers started to increase after 15 December, reaching high ‘breeding season’ numbers around day 50–60 (late February).

**Figure 2:**
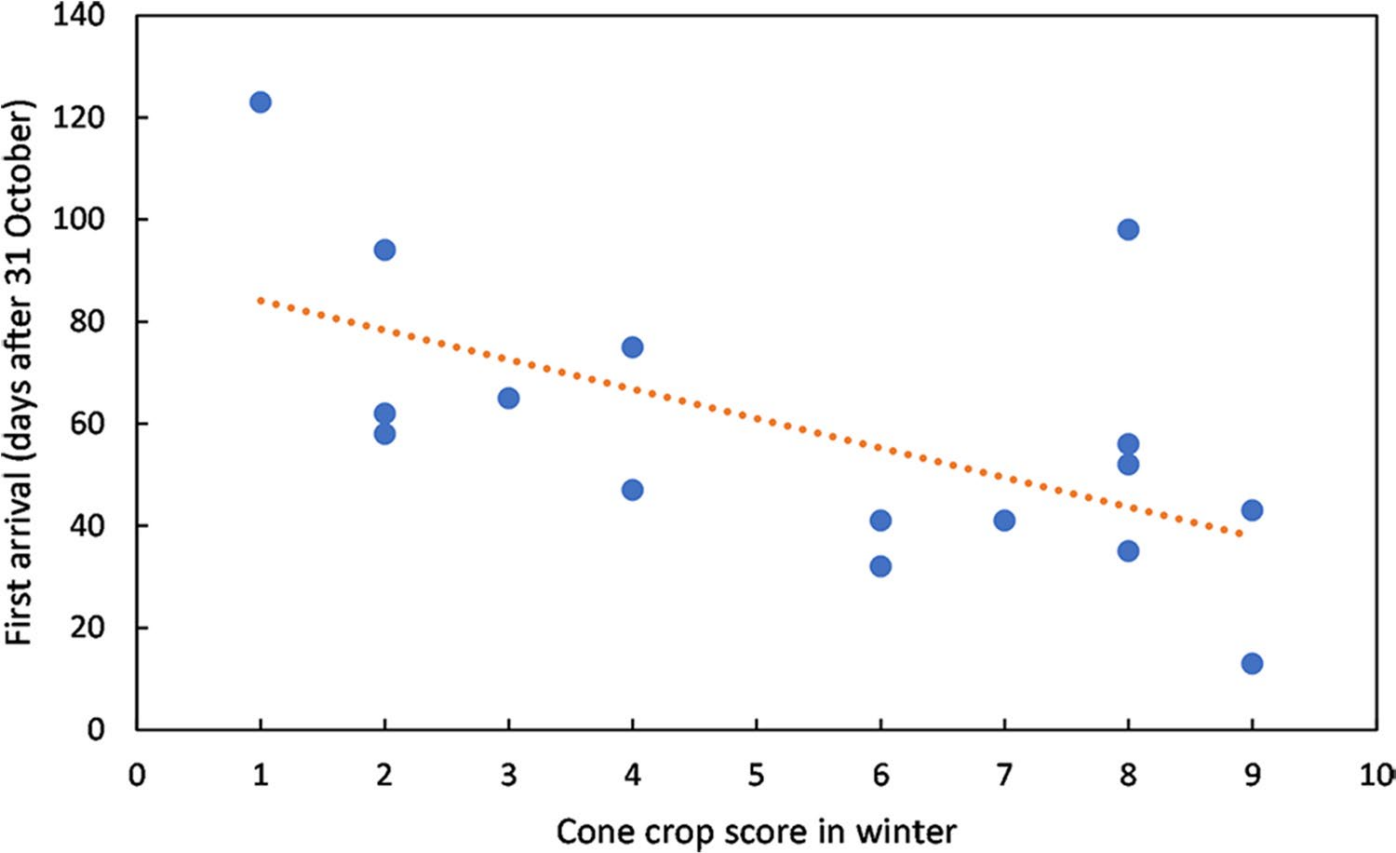
First arrival of siskins. Date of first capture (days after 31 October) in relation to cone crop score that winter. Data for 2005–2020. R^2^ = 0.3433.

**Figure 3:**
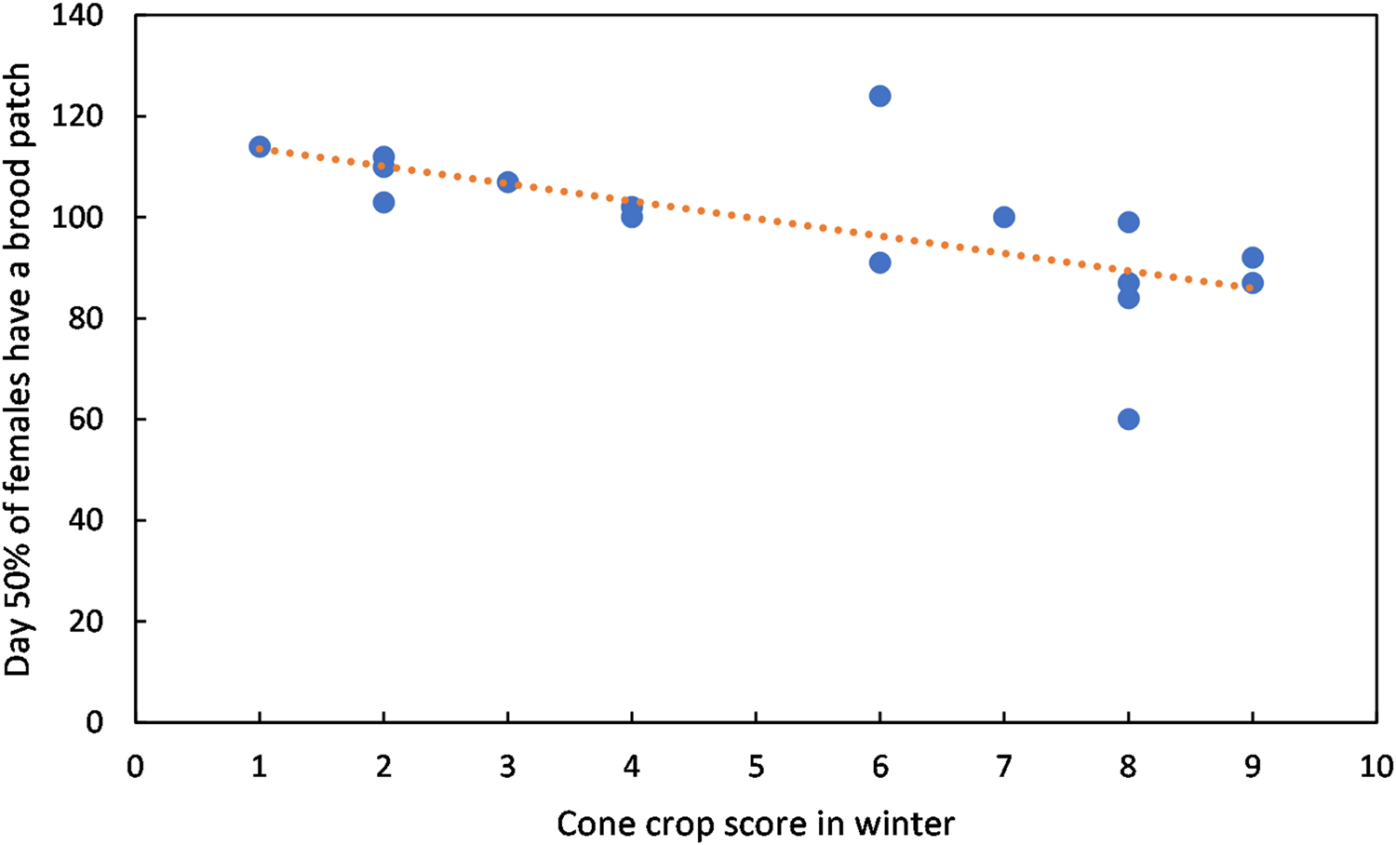
Cone crop impact on timing of brood patch formation. Day of year on which 50% of female siskins had brood patches in relation to cone crop score of previous winter. Data for 2005–2020. R^2^ = 0.4237.

**Figure 4:**
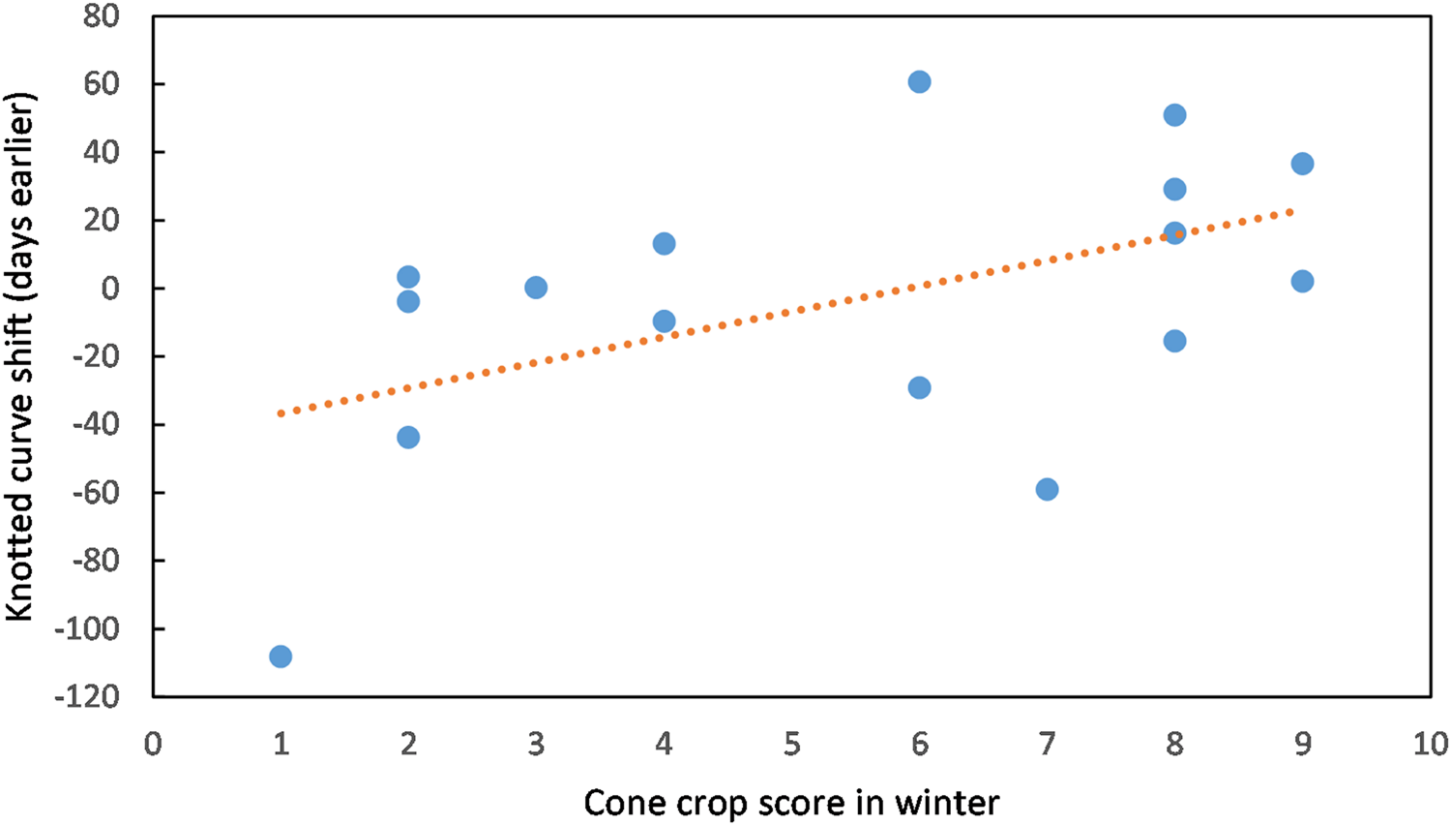
Cone crop impact on timing of sex ratio change. Number of days earlier in the year that changes in sex ratio in catches occurred, as a function of cone crop score of the previous winter. Data for 2005–2020. R^2^ = 0.2485.

Part of the variability in catch numbers can be explained by different times of arrival of siskins in the study area in different years. After several catches with no siskins present in October–November, the date of first capture in each season varied from 13 November in 2011 to 25 February in 2009 (Table 1). The date of first capture, expressed as days after 31 October, showed a significant relationship with the Sitka spruce cone crop score that winter (ANOVA linear regression: F = 7.3186, n = 16, p = 0.0171, Fig. 2), with each one point increase on the cone crop scale equivalent to birds arriving 5.8 days earlier (regression slope =  − 5.8). Using the presence of a well-formed brood patch on female siskins as a proxy for breeding^18^, breeding also occurred earlier in years with a high cone crop (ANOVA linear regression: F = 10.291, n = 16, p = 0.00632, Fig. 3), with 50% of female siskins having a well-formed brood patch 3.5 days earlier for each one point increase on the cone crop scale. The number of adult female siskins captured, as a proportion of all adults, tended to be lower in the breeding season (Supplementary Fig. 1), and the timing of this reduction was significantly correlated with cone score. Knotted function analysis indicated that the curve of number of females, as a proportion of all adults, was shifted an average of 7.5 days earlier per one point increase in cone score (ANOVA linear regression: F = 4.630, n = 16, p = 0.049) (Fig. 4).

**Table 1:** Summary of sample.

Year of ripe cone crop	Sitka cone score at Argyll park	Days mist netting	Siskins caught at Tarbet site	Initial arrival	Days mist netting during siskin abundance	Period of siskin abundance	Correlation between numbers of adults caught and rain (mm) in previous three days (bold = p < 0.05)	Mean adults per catch in period of abundance
2005	8	35	503	6 Feb	19	17 Feb–29 Jul	0.06	11.2
2006	2	31	425	28 Dec	14	6 Apr–5 Aug	0.34	19.4
2007	8	28	607	5 Dec	18	13 Feb–17 Aug	0.37	21.2
2008	6	32	1128	2 Dec	18	21 Jan–15 Jul	− 0.31	43.3
2009	1	38	1005	25 Feb	15	3 Apr–7 Aug	**0.52**	51.5
2010	7	42	1634	11 Dec	29	5 Jan–29 Jul	0.22	48.0
2011	9	28	1470	13 Nov	21	12 Jan–19 Aug	0.35	33.6
2012	4	33	1017	14 Jan	16	13 Mar–15 Aug	0.29	51.8
2013	6	33	605	11 Dec	15	26 Feb–10 Aug	0.18	25.3
2014	4	28	643	17 Dec	13	22 Mar–27 Aug	0.39	33.0
2015	8	33	986	26 Dec	20	6 Feb–15 Aug	0.35	13.8
2016	2	44	1481	1 Jan	22	26 Feb–27 Jul	− 0.23	47.8
2017	3	42	1805	4 Jan	20	6 Mar–23 Jul	0.15	76.6
2018	8	43	1158	22 Dec	21	19 Feb–25 Jul	**0.57**	42.2
2019	2	38	1261	2 Feb	18	25 Mar–8 Aug	− 0.20	48.4
2020	9	40	825	13 Dec	36	4 Jan–8 Jul	**0.63**	24.3

### Fledgling abundance and timing

**Figure 5:**
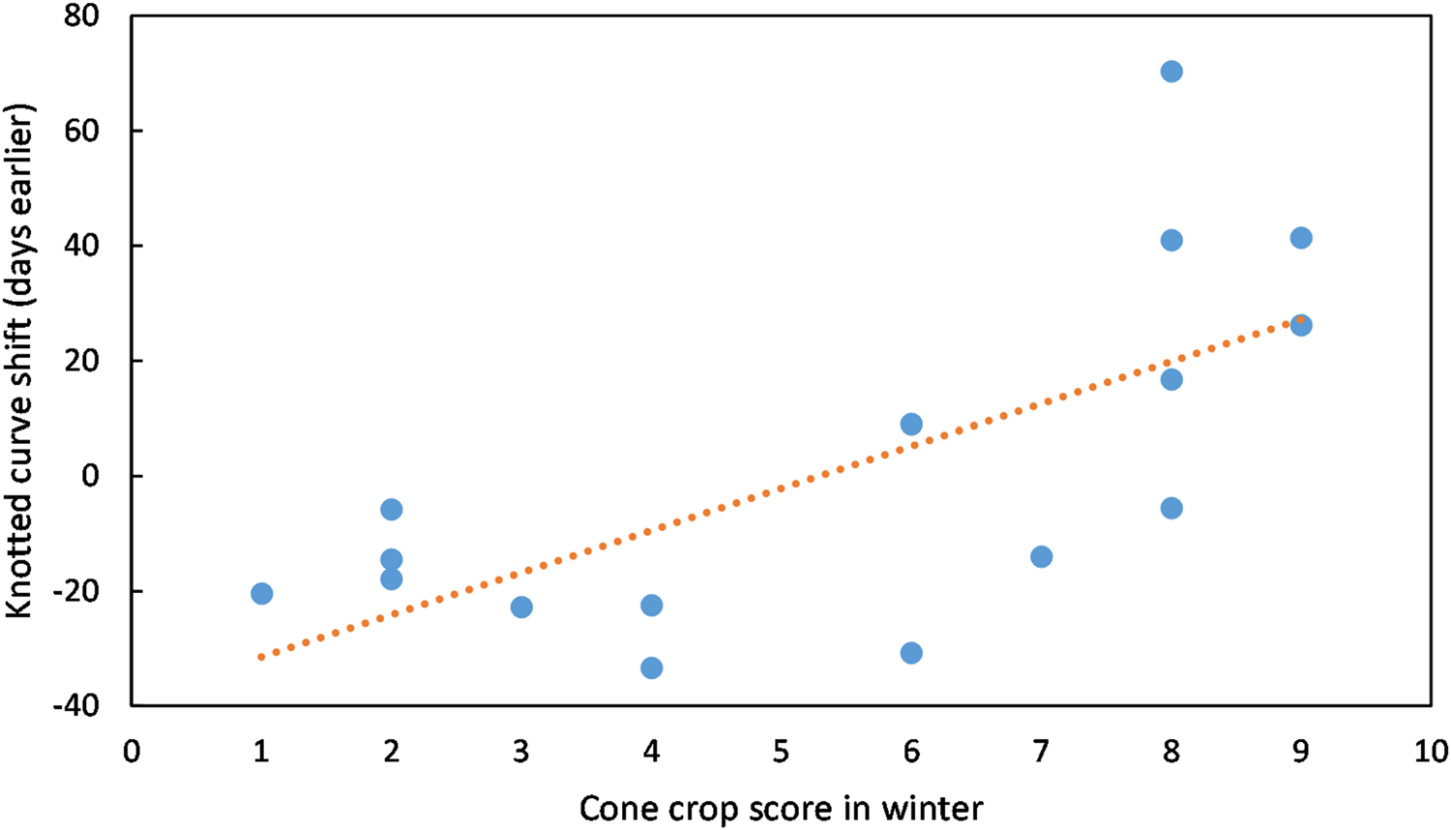
Crop cone impact on timing of fledging. Number of days earlier in the year that the spike in fledgling abundance occurred as a function of cone crop score in the previous winter. Data for 2005–2020. R^2^ = 0.4712.

**Figure 6:**
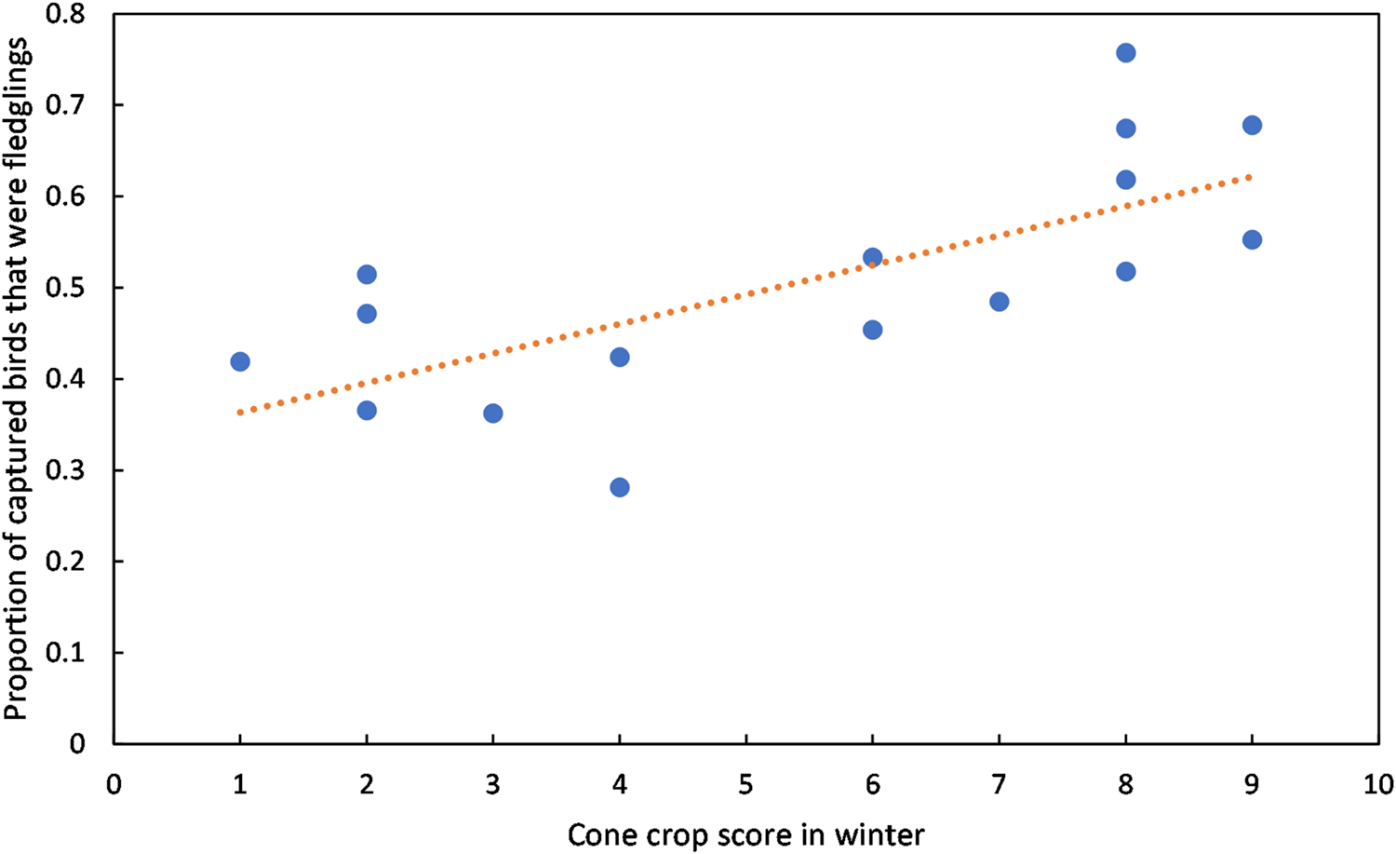
Fledgling abundance by year. Proportion of all captured siskins in June and July identified as fledglings in each year (2005–2020). R^2^ = 0.5082.

The number of fledgling siskins captured, as a proportion of all siskins, increased during the breeding season (Supplementary Fig. 2), and knotted function analysis indicated that the curve of number of fledgling siskins, as a proportion of all siskins, was shifted an average of 7.3 days earlier per one point increase in cone score (ANOVA linear regression: F = 12.473, n = 16, p = 0.003) (Fig. 5). Additionally, the number of fledgling siskins captured at the peak of the breeding season (June and July) (Supplementary Fig. 2), as a proportion of all siskins caught during this period, was higher in years with higher cone scores (ANOVA linear regression: F = 14.465, n = 16, p = 0.002) (Fig. 6). This local result was also supported by a similar trend in the national data: the proportion of siskins ringed each year throughout Britain and Ireland in 2002–2019^24^ that were juveniles was higher in years when our measurement of cone crop score was higher (ANOVA linear regression: F = 10.814, n = 18, p = 0.00463).

### Impact of rainfall

After the increase in siskin numbers during the pre-breeding period, numbers fluctuated throughout the breeding season and then declined after breeding to close to zero (Fig. 1). The timing of the period with consistently high numbers in mist net catches and the mean numbers caught during this period varied among years (Table 1). Rainfall (total amount in mm) in the 3 days before the catch showed a positive relationship with numbers caught in 13 of the 16 years (binomial test of positive rather than negative trend in each year p = 0.012), with the within-year correlation being statistically significant in three of the years (Table 1). Catch numbers thus tended to be higher immediately after a period of wet weather.

### Diet

**Figure 7:**
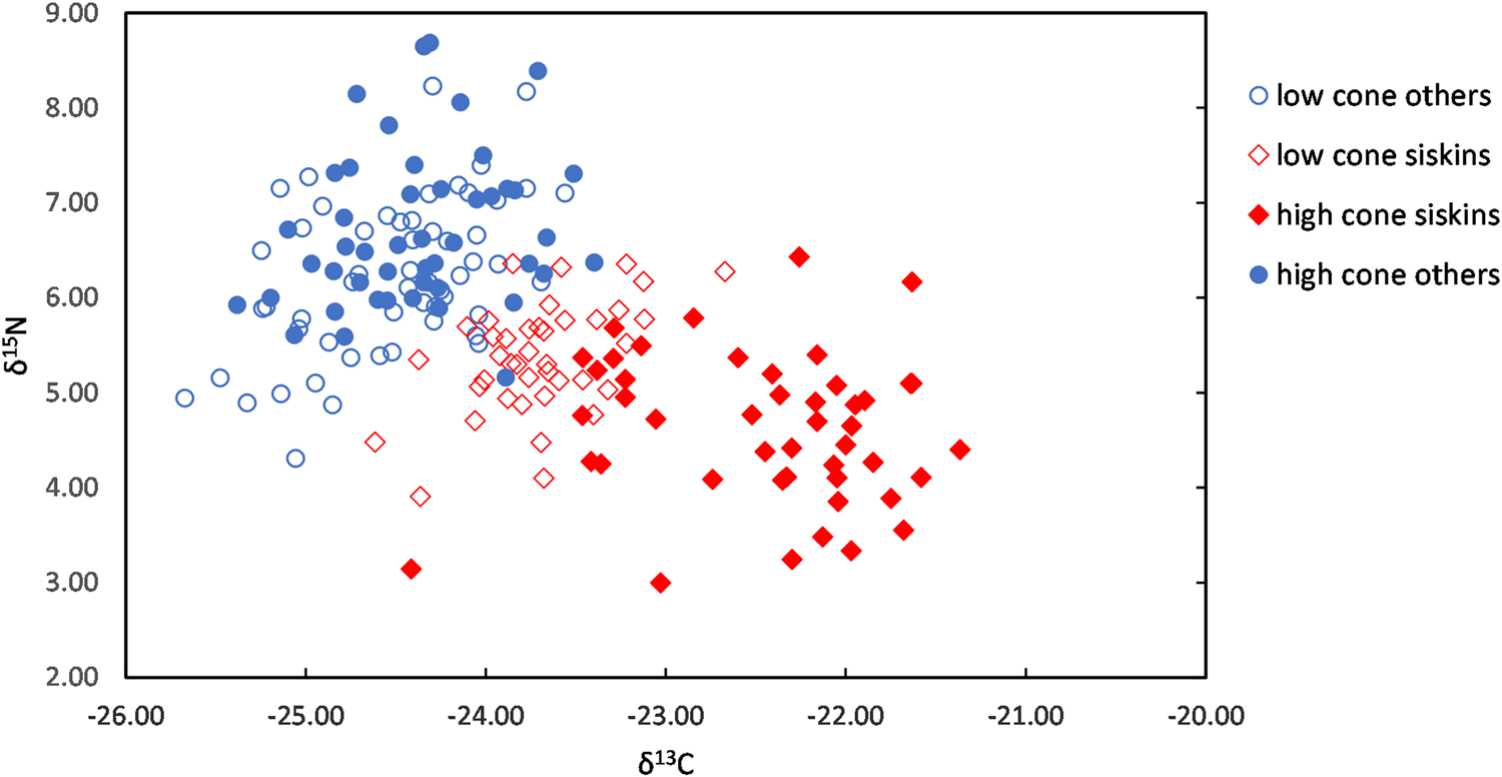
Carbon–nitrogen isotope biplot of each tested sample. Siskins in red, all other birds in blue. High cone crop (score ≥ 6) years are filled symbols low cone crop (score < 6) years are open symbols.

Analyses of the carbon and nitrogen stable isotope ratios in feathers from fledgling siskins and other small passerine fledglings showed that siskins had a distinctly different isotopic fingerprint from the other species (chaffinch *Fringilla coelebs*, goldfinch *Carduelis carduelis*, blue tit *Cyanistes caeruleus*, great tit *P. major*, coal tit *Periparus ater*, willow warbler *Phylloscopus trochilus*, robin *Erithacus rubecula*, dunnock *Prunella modularis*, blackbird *Turdus merula*; Fig. 7). Δ^15^N was significantly lower in siskins compared with other species (Welch two-sample t-test: t_188_ = 12.36, p < 2.2 × 10^–16^), indicating a diet at a lower trophic level. The δ^15^N of Sitka spruce seeds was 0.09‰ and the diet to feather fractionation of ^15^N is about 4‰^25^, and feathers of birds fed exclusively on Sitka spruce seeds would thus be expected to have a δ^15^N of 4.09‰. Siskin fledgling feathers had a δ^15^N of 4.97‰ (n = 86, standard deviation (s.d.) = 0.78), suggesting a diet dominated by seeds rather than by insects. However, δ^13^C was also significantly different (less depleted) compared with other species (Welch two-sample t-test: t_188_ = 13.931, p < 2.2 × 10^–16^), indicating a strong difference in the diets of siskin and other passerine chicks.

**Figure 8:**
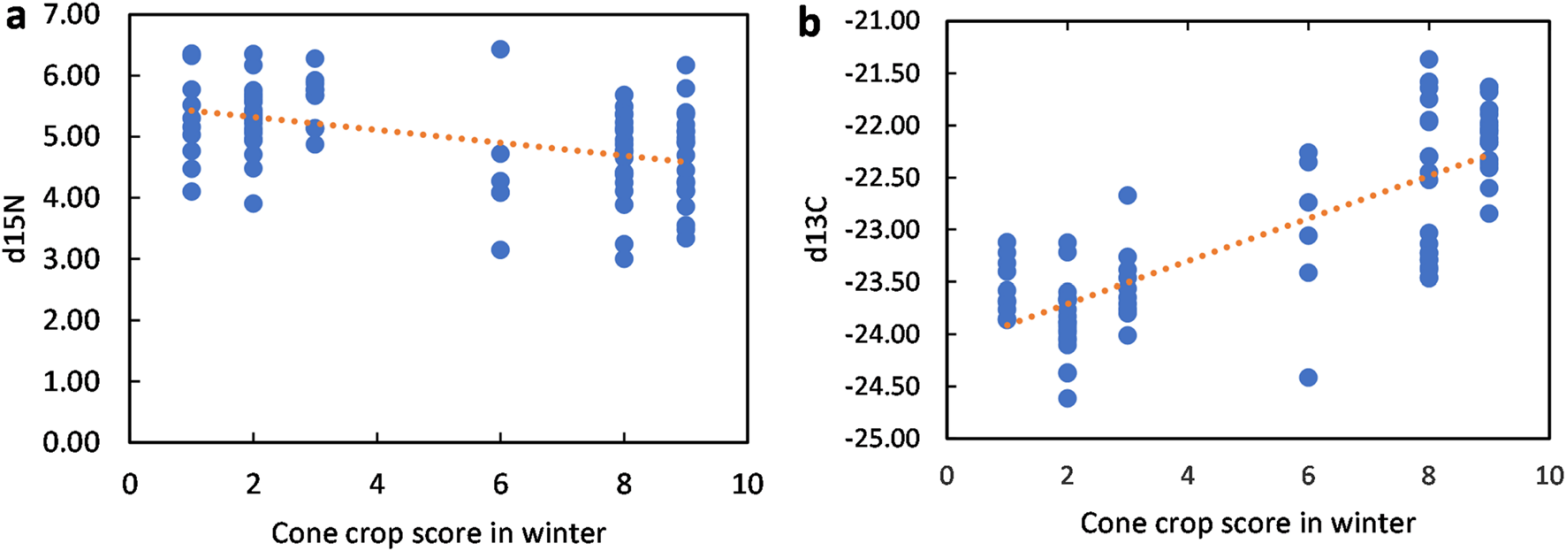
Impact of cone crop on nitrogen and carbon stable isotopes. (**a**) Nitrogen isotope ratio of siskin fledgling feathers in relation to cone score. R^2^ = 0.1868. (**b**) Carbon isotope ratio of siskin fledgling feathers in relation to cone score. R^2^ = 0.6192.

Siskin fledglings had significantly lower δ^15^N values (ANOVA linear regression: F = 19.291, n = 86, p = 3.252 × 10^–5^, Fig. 8a) and significantly higher δ^13^C values (ANOVA linear regression: F = 136.6, n = 86, p < 2.2 × 10^–16^, Fig. 8b) in years with higher cone scores. The isotopic ratios in years of high cone crop (cone score >  = 6) (mean δ^15^N 4.8‰ and mean δ^13^C − 22.4‰) were close to those predicted from the isotope ratios for birds fed exclusively on Sitka spruce seeds (δ^15^N 4.1‰, δ^13^C − 24.4 to − 19.5‰). In chaffinch fledglings (the largest sample size for a different species), δ^15^N increased significantly with cone score [ANOVA linear regression: F = 5.9872, n = 35, p = 0.0199, mean during low cone score years 6.37‰ (n = 20) and mean during high cone score years 6.86‰ (n = 15)], although δ^13^C did not change significantly (ANOVA linear regression: F = 1.5972, n = 35, p = 0.2152, mean − 24.2‰). Similarly, δ^15^N increased significantly with cone score (ANOVA linear regression: F = 4.0583, n = 67, p = 0.0481), and δ^13^C did not change significantly with cone score (ANOVA linear regression: F = 0.8078, n = 67, p = 0.3721) in fledglings of all other small passerine species combined (excluding chaffinch). In years of very low cone crop, the δ^13^C for siskin fledglings was more similar to that for chaffinch and other small passerine fledglings, at around − 24‰ in siskin (Fig. 7).

### Adult abundance

The mean number of adult siskins caught per day during the breeding season showed a slight sign of increase from 2005 to 2020, from about 30 to about 45 birds per catch (Table 1). As such, analysis of the impact of cone crop on adult catch rate was performed with year as a covariate, in order to control its effect. The mean number of adult siskins caught per day was significantly lower in years with higher cone crops (ANOVA linear regression, with “year” as an additive covariable: F = 5.6912, n = 16, p = 0.03296).

### Juvenile weight and size

Monthly mean weight of newly fledged juvenile siskins tended to increase over the course of the breeding season (ANOVA linear regression, additive model containing cone score and month (May–July), excluding months with < 10 captures: F = 17.3465, n = 37, p = 7.104 × 10^–6^), as did monthly mean juvenile wing length, although the latter relationship is very weak (ANOVA linear regression, additive model containing cone score and month (May–July), excluding months with < 10 captures: F = 3.3847, n = 37, p = 0.04602) and data from a wider range of months suggest that this trend may not be meaningful (Supplementary Table 1). Cone score had no significant impact on either of these measurements (ANOVA linear regression, additive model containing cone score and month (May–July), excluding months with < 10 captures: F = 0.5605, n = 37, p = 0.4594) and (ANOVA linear regression, additive model containing cone score and month (May–July), excluding months with < 10 captures: F = 0.0373, n = 37, p = 0.84801) respectively.

### Carry-over effects

There was no significant relationship between the number of adult siskins born in the previous calendar year, as a proportion of all adult siskins, and the cone score in the previous year (ANOVA linear regression: F = 0.004, n = 16, p = 0.949).

### Cone ripening

In 2006–07, the cumulative percentage of seeds shed from cones began to increase as cones ripened in late September (Supplementary Fig. 3). The percentage shed in autumn increased more rapidly in Perthshire than in Argyll, and a higher percentage of seeds were shed throughout the winter, spring and summer in Perthshire compared with Argyll. However, some seeds remained within the cones throughout the siskin breeding season (i.e. until the end of July) in both sites. About 10% of seeds were still in the cones at the end of July 2007 at the Argyll site, when siskins left the area (300 days after 1 October).

## Discussion

Siskins are generally thought to move south from their breeding areas in Scotland to spend the autumn and early winter in southern Britain or southern Europe^3,11^. Siskins in the current study were accordingly generally absent from mist net catches at the study site in late summer and autumn. However, siskins started to return to the study area from as early as mid-November/December in years with high Sitka spruce cone crops, with the return starting 5.8 days earlier for each point increase in the cone crop index. Shaw^18^ showed that siskin breeding occurred earlier in years of high cone abundance, based on a small number of years in the 1980s. Using the same method of determining timing of breeding as Shaw (presence of brood patches on females), breeding started 3.5 days earlier for each one point increase in cone crop index, while using novel methods of determining timing of breeding, including the reduced proportion of adult females in catches and increased numbers of fledglings in catches, breeding started 7.5 and 4.8 days earlier for each one point increase in cone crop index, respectively. Although birds return to the study area to breed, date of return and date of breeding do not need to be correlated, as birds may spend different amounts of time before being able to gather resources required to form eggs, depending on food availability in different years. The earlier return of birds in years of high cone crop is a novel finding, and may partly explain why numbers of siskins ringed in southern England vary so much from year to year^24^: siskins may spend less time in southern England during late winter and spring in years with large cone crops, and instead return to their breeding areas in Scotland to exploit the large cone crop. However, if this behaviour occurs, the mechanism that controls it is not clear. If siskins are highly mobile in late winter, they might discover abundant Sitka spruce seeds in their breeding areas and aggregate there early, or alternatively, they might assess the size of the forthcoming cone crop in late summer as it begins to ripen, and thus commit to returning earlier or later the next year based on this assessment. However, there is currently no evidence to support either of these hypotheses. Although it has been suggested that some siskins are nomadic and may breed in different locations in successive years^11^, some individuals are highly site faithful: e.g. the same bird was caught at our study site in eight of the 10 breeding seasons from 2010 to 2019. A detailed analysis of the national ring recovery data might shed further light on the extent of seasonal movements and thus help to explain the variation in phenology in relation to cone crop, and the extent of nomadic versus site-faithful behaviour.

Petty et al.^26^ found 14 siskin territories per hectare in Sitka spruce plantations in Kielder, Northumberland, in early and late spring 1991, a year of very high Sitka spruce cone crop, but only 0.5 and three territories per hectare in early and late spring respectively in 1992. Given that the size of the Sitka spruce cone crop is synchronous across the whole of Britain^13^, it is unlikely that Sitka spruce plantations elsewhere in Britain would have offered better resources for siskins in 1992, and it thus seems likely that siskins either nested in other habitats or refrained from breeding because of the poor Sitka spruce cone crop. However, there is currently little evidence to suggest that siskins breed in large numbers in Britain in any habitat other than conifer forest^3^, but there is also very little evidence of nonbreeding by siskins in poor cone crop years. Virtually all adult female siskins caught in our study in May and June had a fully developed brood patch (as an indication of breeding activity^27^), even in years with a poor Sitka spruce cone crop. Siskins clearly started to breed later in the spring in poor cone crop years, possibly resulting in fewer pairs having a second brood because there would be less time available before the seed resource was depleted and the birds thus needed to leave the area to find other food resources. Although siskins are considered to be nomadic and to search for alternative patches with food^18^, it remains unclear if large numbers of siskins leave the breeding area in years of poor cone crop and refrain from breeding at all, since the synchronous masting of Sitka spruce across the whole of Britain^13^ suggests that no alternative breeding opportunities would be available elsewhere.

Stable nitrogen isotope ratios showed that siskin chicks were fed at a lower trophic level than chicks of other small passerine species in the study area. Furthermore, the ratios of stable nitrogen and carbon isotopes were both consistent with siskin chicks being fed almost exclusively on Sitka spruce seeds during years of high cone crop, but having a diet composition more similar to that of chicks of other small passerine species in years with a poor cone crop. This poor cone crop diet has a higher δ^15^N, which implies feeding at a higher trophic level than in years of high cone crop^19^. Consequently, despite the fact that the poor cone crop diet δ^13^C is still marginally within the range of values predicted for a diet consisting purely of Sitka spruce seeds, such a diet in years of poor cone crop would be incongruent with both the observed change in trophic level, and the predictable change in δ^13^C observed in those years. Consistent with the importance of Sitka spruce seeds in the siskin diet during the breeding season, a poor cone crop (Supplementary Fig. 4) and wetter weather (Table 1) (causing the cones to remain closed) were associated with higher catches of siskins at the study site between the plantation forestry and other habitats, likely as a result of their moving out of the plantation to look for alternative food resources. The numbers of seeds remaining in cones on the trees showed that relatively few seeds were shed in autumn or winter, and seeds were shed faster from cones in Perthshire than in Argyll. This is consistent with studies in Alaska, which reported lower seed fall in more humid locations and lower seed fall during winter than in autumn or spring^7,8^. It is also consistent with observations on Sitka spruce seed fall in Speyside^10^, which showed that some seeds remained in cones throughout the summer. The results of the current and previous studies^7,8,10^ indicate that cones may be suitable for siskins to exploit throughout their breeding season, while the departure of siskins from their breeding areas in Scotland in late summer may coincide with a drop in seed density to below a level that is economically exploitable by siskins. Timing of breeding and the end of the breeding season in common crossbills *Loxia curvirostra* have also been attributed to seed fall from Sitka spruce cones, which makes foraging less productive as spring progresses^28^. Stable nitrogen isotope ratios showed that chicks of other species, including chaffinches, were fed at a higher trophic level in years of high cone crop than in years of poor cone crop. This was unexpected, but could be the result of niche contraction^29^ by non-siskins in years of higher cone crop, in response to increased foraging by siskins depleting lower trophic-level food sources in these years. If this is the case, then it suggests that competition with siskins has a substantial impact on the diet of other small passerines.

Given that siskins exploit Sitka spruce seeds as a food source, especially in years with large cone crops, it is reasonable to expect an increase in biomass of fledgling siskins in high cone-crop years. This increased biomass could be reflected by an increase in the size and weight of individual chicks, or by an increased number of fledged chicks, or both. We found no evidence of any differences in fledgling size or weight in relation to cone score. This is perhaps unsurprising given that the body weights and sizes of birds are known to be regulated by a variety of factors, including predation^30^. However, we found evidence of an increase in the number of fledglings in years with larger cone crops, suggesting that siskin breeding success was limited by food availability. Our local finding was also consistent with the national ringing data, as the total number of juvenile siskins ringed in Britain and Ireland each year was a higher proportion of the annual catch in years with a larger cone crop.

Siskins start to breed in their second calendar year^5^. It was therefore puzzling to note that the age distribution of adult siskins did not reflect the productivity of the previous year. Shaw^18^ recaptured 33 adult siskins in subsequent years that had previously bred in his study area and had returned, but only one juvenile, suggesting very much higher dispersal of juveniles. However, even if juvenile dispersal is very high, the fact that Sitka spruce cone crop is synchronous across the whole UK^13^ suggests that the proportion of 1st year birds in catches should reflect national breeding output from the previous year. If overwintering deaths of siskins occurred at random, then we would expect to see a greater proportion of 1st year adults in the breeding season following a large cone-crop year; however, this was clearly not the case. One possibility is that overwintering deaths of birds are not random, but are biased towards juveniles. Coupled with density-dependent winter mortality, this could eliminate any effect of cone crop on the age distribution of breeding adults. This would not be a surprising conclusion: juvenile birds differ from adults physically^31^ and are clearly less experienced than adults, potentially leading to increased mortality under conditions where resources are scarce, such as during the winter^32^. In line with this hypothesis, Newton (1989)^33^ estimated that about 70–85% of fledglings of a range of bird species died before reaching breeding age, compared with significantly lower annual adult mortality.

Climate change models predict drier summers and wetter winters in the north and west of Scotland^34^, which comprises the core breeding range for siskins in Britain and Ireland. Climate change may influence masting^14–17^, and may also affect management decisions for plantation forestry^35–37^. Given the current large areas of Sitka spruce plantations, such changes are likely to result in reduced Sitka spruce coverage and, consequently, reduced availability of Sitka spruce seeds. A projected decline in mature conifer plantations in Britain^38^ may thus lead to a decline in siskin numbers over the coming decades. However, changes in forestry practice to encourage natural regeneration and an increase in more diverse open woodlands managed for biodiversity and amenity (a more open woodland structure being required to allow people to walk through) as well as timber production^36^, may provide increased cone crops despite an overall reduction in the number of Sitka spruce trees, because less crowded and older trees produce many more cones than trees planted at high density^39^. Although the current stable isotope results indicated that siskins can switch from feeding on Sitka spruce seeds to alternative diets when the cone crop is poor, larger cone crops clearly encourage breeding and increase siskin productivity. The relationship between siskin numbers caught in mist nets and preceding periods of rainfall also suggest that siskins can adjust their foraging activity in relation to short-term changes in seed availability. The lack of any effect of the previous year’s cone crop on the age structure of adult siskin populations implies the existence of a bottleneck in siskin population sizes outside the breeding season, presumably in winter. It would therefore be worth investigating ring recovery data to determine if siskin survival appears to be influenced by cone crop, thus allowing an assessment of how resilient the population might be to the effects of climate change.

The details that we present here surrounding the response of siskins to environmental forcings have broader implications, particularly for conservation. Siskins in the UK are vastly more abundant in the modern day than they were in the nineteenth century^3,4^, a phenomenon that can be attributed to the widespread planting of Sitka spruce in the twentieth century^3,13^. This presents an excellent example of an interesting and underexplored phenomenon in ecology: ecological switching by a native species to reliance upon a non-native. This phenomenon comes with certain conservation implications, especially in cases where the original niche of the native species has declined in availability, and this phenomenon is likely to be observed more frequently in future given the increased prevalence of non-native species as major components in ecosystems worldwide. While other examples exist where this phenomenon has been observed^40–42^, this is, to our knowledge, the most extensive study of such a relationship, and should provide an excellent point of comparison for other equivalent scenarios.

## Methods

### Study site

Small passerines were caught using a 13 m mist net placed between trees and shrubs on the edge of the village of Tarbet, Argyll & Bute (56.21 N 4.71 W), adjacent to a large forestry plantation. Siskins can forage up to at least 5 km from their nest during the breeding season^5,18^, and the area within 5 km was therefore considered likely to include breeding siskins that would move through the catching site. Plantation forestry species, age, and area were determined from maps from the Forestry Commission compartment data base. There were 1152 ha of plantation forestry within 5 km of the catching site, comprising 79% Sitka spruce, 7% Norway spruce *Picea abies*, 13% larch and < 1% Scots pine *Pinus sylvestris* and lodgepole pine *Pinus contorta*. Most of the area was planted between 1952 and 1976, and most of the Sitka spruce was therefore of cone-bearing age when the study started in 2005^36^. Tree age has been shown to have a strong influence on seed production, even in mature trees^17^, and given that very little felling occurred in the area up to 2020, cone production was likely to have increased throughout the study period (2005–2020). Other major habitats within this radius include freshwater (Loch Lomond), ancient woodland dominated by oak, regenerating woodland dominated by birch, open moorland with scrub, and rural village gardens. Deciduous woodland and rural village gardens may provide alternative foraging opportunities for siskins, and in particular many gardens contain bird feeders providing seeds and peanuts, which can be used by siskins and some other small passerines^12^ and so may attract siskins and other birds. A total of 12,513 ha of plantation forestry (76% Sitka spruce) was located within a wider radius (20 km) from the catching site, with about 2500 ha planted each decade from 1950 to 2000. Plantation forestry is thus an important habitat both locally and over the wider area. Daily rainfall data were provided by the Met Office from a weather station 4 km north of the study site (Sloy Power Station, Inveruglas, 56.25 N 4.71 W).

### Cone scores and seed shedding

Sitka spruce cone production was assessed using a 10-point scale developed by Petty et al.^26^. The score was based on a visual assessment of the relative abundance of ripe cones in late autumn/early winter throughout the forestry plantation. Cone crop was scored from zero (no cones visible on the tree canopy when viewed with binoculars from a vantage point) to 10 (exceptionally large crop, the entire upper tree canopy appearing brown with cones when viewed from vantage points). This is essentially the same scale used by Shaw^18^ in SW Scotland, but divided into 10 rather than five categories. Scoring was generally carried out over a few days in autumn before the cones had fully ripened, to discount any remaining old cones from the previous years, by viewing through binoculars all of the areas of plantation forestry from overlooking vantage points. Bird data were related to the cone crop in the preceding autumn/winter, because seeds remain within the cones for up to 12 months^10^, and the breeding of siskins in year X would therefore be expected to be influenced by the cone crop in year X − 1. The cone score was only recorded for Sitka spruce as the main conifer in the study area, but the Norway spruce cone crop has been shown to correlate with the Sitka spruce crop, and cone crop is synchronous across the whole of the UK^13^, so the cone scores derived in this study apply also to other parts of the UK.

In order to assess whether Sitka spruce seed was available to siskins as food throughout their breeding season, we carried out a cone ripening study to measure seed loss from cones. Seed retention was estimated by sampling Sitka spruce cones (one per tree) from a sample of 10–30 trees at approximately 1-month intervals from 1 October 2006 to 31 July 2007 (a fairly typical year in terms of rainfall according to Met Office data but a year with high cone crop) from two locations, Argyll Forest Park near Tarbet (within 1 km of the bird-catching site) and West Lamberkine Wood, Perthshire. The latter site was chosen to represent an area of Scotland with generally drier weather conditions compared with Argyll, to determine if seeds were shed faster in a drier climate, as found in Alaska^7,8^. Individual cones were cut using a 4 m pruning pole and handled carefully to avoid seed loss due to cutting. Cones were stored individually in polythene bags in the field and then placed in a dry room for several days to allow the cones to open. The seeds were then extracted by tapping the cones over a sheet of paper, and the number of scales per cone and the number of extracted seeds were counted. Each scale initially holds two seeds, and the maximum number of seeds was calculated as: (number of scales) × 2. The number of seeds extracted was then expressed as a percentage of the maximum number of seeds. Monthly sampling of cones was not carried out in other years, so we cannot be certain that seed loss is the same in years of low cone crop.

### Catching and processing birds

Mist netting was carried at intervals of about one week. However, as mist netting can only be done at low wind speeds and under dry conditions, the interval between catches varied according to the weather conditions. Mist netting was carried out on a total of 568 days between 2005 and 2020, with annual totals of 28–44 days (Table 1), resulting in the capture of 50,282 birds, including 16,553 siskins. The mist net was kept under continuous observation and birds were normally removed from the net as soon as they were caught. On a few occasions, the net had to be closed to prevent a build-up of birds if the catch rate was exceptionally high or because weather conditions changed (usually rain showers), but catching was usually continued throughout the day, from about 0700 to about 1600 GMT. Each bird was identified to species, ringed (or its ring number was recorded if had been ringed previously), classified according to sex when possible^43^, weighed (to the nearest 0.01 g) using an electronic balance, and wing length (maximum chord) was measured (to the nearest mm). The time of capture was noted to the nearest 30 min. Age of siskins was determined based on the shape of the tail feathers and the presence or absence of a colour contrast between the inner and outer greater coverts^43^. Adult female siskins were checked for brood-patch score (as in Shaw 1990^18^) and adult siskins were checked for primary moult score^27^. Any feathers shed by juvenile birds caught before their post-juvenile moult (i.e. in the short period immediately following fledging), or any tail feathers shed by juveniles were stored dry in a labelled paper envelope for stable isotope analysis. All birds were released, usually within a few minutes after initial capture. Mist net capture and ringing of birds was carried out under the supervision of R.W.F., British Trust for Ornithology (BTO) bird ringing permit number S/2282, following BTO guidelines and regulations.

### Stable isotope analysis

Sitka spruce seeds were sampled from cones in Argyll Forest Park in 2005, 2006, 2017, 2018, 2019 and 2020. Pooled samples of seeds from about 30 cones were used for stable isotope analysis, with approximately equal representation of each of the years in which seeds were sampled. Seed samples were milled to a fine powder in a Retsch MM200 ball mill before weighing. Ground seed samples (1.5 ± 0.1 mg) were weighed into tin capsules (8 × 5 mm). Up to 10 samples of feathers were collected from different juvenile siskins from each year for which feathers were available: six from 2001, and 10 from each of 2007, 2009, 2011, 2016, 2017, 2018, 2019 and 2020 (86 birds in total). Feathers from other juvenile small passerines that had not yet entered post-juvenile moult were sampled for the same years: 35 chaffinches, 21 coal tits, 10 blue tits, nine great tits, eight robins, six blackbirds *Turdus merula*, six dunnocks, six goldfinches and two willow warblers. Feather samples were prepared by finely chopping with surgical scissors and 1 ± 0.1 mg samples were weighed into tin capsules (8 × 5 mm).

Carbon and nitrogen isotope ratios of feather and seed samples were analysed by elemental analysis—isotope ratio mass spectrometry. Samples and reference materials were weighed into tin capsules, sealed, and loaded into an auto-sampler on a Europa Scientific elemental analyser. Combusted gases were swept in a helium stream over a combustion catalyst (Cr_2_O_3_), copper oxide wires (to oxidize hydrocarbons), and silver wool to remove sulphur and halides. The resultant gases were swept through a reduction stage of pure copper wires held at 600 °C_._. Water was removed using a magnesium perchlorate chemical trap. Nitrogen and carbon dioxide were then separated using a packed column gas chromatograph held at a constant temperature of 65 °C. The resultant nitrogen peak entered the ion source of the Europa Scientific 20–20 IRMS and was ionized and accelerated. Nitrogen gas species of different masses were separated in a magnetic field and measured simultaneously using a Faraday cup collector array to measure the isotopomers of N_2_ at 28, 29 and 30 *m/z*. After a delay, the carbon dioxide peak entered the ion source and was ionized and accelerated, and carbon dioxide gas species of different masses were separated in a magnetic field and measured simultaneously using a Faraday cup collector array to measure the isotopomers at 44, 45 and 46 *m/z*. Both references and samples were converted to N_2_ and CO_2_ and analysed using this method. The analysis was carried out in a batch process involving analysis of a reference, followed by several samples and then another reference. The reference material used for δ^13^C and δ^15^N analyses of feather and seed samples was IA-R068 (soy protein, δ^13^CV-PDB =  − 25.22‰, δ^15^NAIR = 0.99‰). IA-R068, IA-R038 (L-alanine, δ^13^CV-PDB =  − 24.99 ‰, δ^15^NAIR =  − 0.65‰), IA-R069 (tuna protein, δ^13^CV-PDB =  − 18.88‰, δ^15^NAIR = 11.60‰) and a mixture of IAEA-C7 (oxalic acid, δ^13^CV-PDB =  − 14.48‰) and IA-R046 (ammonium sulphate, δ^15^NAIR = 22.04‰) were run as quality control samples. IA-R068, IA-R038 and IA-R069 were calibrated against and traceable to IAEA-CH-6 (sucrose, δ^13^CV-PDB = − 10.449‰) and IAEA-N-1 (ammonium sulphate, δ^15^NAIR = 0.40‰). IA-R046 was calibrated against and traceable to IAEA-N-1. IAEA-C7, IAEA-CH-6 and IAEA-N-1 were inter-laboratory comparison standards distributed by the International Atomic Energy Agency, Vienna. These are standard methods for stable isotope analysis, and are similar to those described elsewhere^44,45^.

### Data analysis

Analysis was performed using the dataset of birds caught from 2005 to 2020. Although birds were captured in previous years, ringing was less frequent and the sample sizes were smaller. Linear regression analyses were performed in R^46^ and Excel^47^, and ANOVA analyses were performed in R^46^ with continuous predictors (except in the case of ‘month’ in the analysis of fledgling siskin weights and wing lengths, which was a categorical variable). Siskin adult and juvenile annual ringing totals for Britain and Ireland for 2005–2019 were extracted from Robinson et al.^24^.

We used several metrics of timing of breeding. Numbers of adult females caught was used as one index of timing of breeding because female siskins incubate eggs whereas males do not, so when incubation starts the proportion of females in catches declined. Once chicks have hatched, the proportion of adult females increases again, so this metric indicates timing of breeding, as does the presence of a brood patch on adult females (which develops as eggs are laid but re-feathers after breeding, whereas adult males do not develop a brood patch). Arrival of fledged young in mist net catches also indicated timing of breeding.

Knotted function analysis was used to fit a curve to certain variables, and differences in the shapes of the curve between years could be quantified to measure changes in the behaviour of the siskin population between years. The curves were fit using the ‘get_natural_cubic_spline_model’ function in the Basis Expansions Python module^48^ within a custom Python function (Supplementary Eq. 1), within a custom Python script (Supplementary Eq. 2). After fitting the curve to the data, the script adjusted the position of the curve independently for each year to minimize the misfit between the predicted and observed values in each year, and recorded the magnitude and direction of shift in each year as a metric of the ‘earliness’ or ‘lateness’ of the siskin population dynamics in that year. Knotted function analysis excluded captures with < 15 valid birds on the basis that captures with few individuals might contain relatively unbalanced sex or age ratios purely by chance. Birds caught after the 220th day of the year were also excluded on the basis that siskins became very rare around this time each year, and the graph-fitting process would therefore be unreliable. This filtering left 208 data points to construct the fitted curve of sex ratios, and 260 data points to construct the fitted curve of age ratios.

Filtering of data and conversion from the records of individual birds into records of individual ringing sessions involved the use of three custom Python scripts (Supplementary Eqs. 3–5): Script 3 was used to analyse the frequency of fledgling siskins, relative to all siskins; Script 4 was used to analyse the frequency of adult female siskins, relative to all adult siskins; and Script 5 was used to analyse the frequency of 1st year adult siskins relative to all adult siskins. The number of juvenile siskins, relative to all siskins, in June and July was analysed by sorting the list of relevant birds by age and counting the number of captures in each category. The proportion of juvenile and/or female siskins were measured rather than absolute numbers in order to remove any influence of variable ringing effort or weather on catch.

## Supplementary Information

Supplementary Dataset 1.

Supplementary Dataset 2.

Supplementary Dataset 3.

Supplementary Information.

## Data Availability

Raw data and python scripts used in this paper are available as Supplementary information.
