# Supplementary material for: Effects of Sitka spruce masting on phenology and demography of siskins Spinus spinus
Source: Sci Rep. 2021 Mar 1;11:4921. doi: 10.1038/s41598-021-84471-8 (PMC7921583; doi:10.1038/s41598-021-84471-8)
Supplement: Supplementary file 4 — Supplementary Information. [file 41598_2021_84471_MOESM4_ESM.docx]

**Title: Effects of Sitka spruce masting on phenology and demography of siskins *Spinus spinus***

**Euan N. Furness^1^* & Robert W. Furness^2^**

1 Science and Solutions for a Changing Planet DTP and Department of Earth Sciences and Engineering, Imperial College London, South Kensington Campus, London SW7 2AZ, United Kingdom

2 Institute of Biodiversity, Animal Health and Comparative Medicine, University of Glasgow, Glasgow G12 8QQ, U.K.

*email: e.furness19@imperial.ac.uk

**Supplementary Dataset File 1**

Ringing data for Tarbet 2005-2020.

**Supplementary Dataset File 2**

Cone crop data.

**Supplementary Dataset File 3**

Stable isotope analysis report.

**Supplementary Equations 1**

import numpy as np

import pandas as pd

from sklearn.base import BaseEstimator, TransformerMixin

from sklearn.linear_model import LinearRegression

from sklearn.pipeline import Pipeline

def get_natural_cubic_spline_model(x, y, minval=None, maxval=None, n_knots=None, knots=None):

"""

Get a natural cubic spline model for the data.

For the knots, give (a) `knots` (as an array) or (b) minval, maxval and n_knots.

If the knots are not directly specified, the resulting knots are equally

space within the *interior* of (max, min). That is, the endpoints are

*not* included as knots.

Parameters

----------

x: np.array of float

The input data

y: np.array of float

The outpur data

minval: float

Minimum of interval containing the knots.

maxval: float

Maximum of the interval containing the knots.

n_knots: positive integer

The number of knots to create.

knots: array or list of floats

The knots.

Returns

--------

model: a model object

The returned model will have following method:

- predict(x):

x is a numpy array. This will return the predicted y-values.

"""

if knots:

spline = NaturalCubicSpline(knots=knots)

else:

spline = NaturalCubicSpline(max=maxval, min=minval, n_knots=n_knots)

p = Pipeline([

('nat_cubic', spline),

('regression', LinearRegression(fit_intercept=True))

])

p.fit(x, y)

return p

class AbstractSpline(BaseEstimator, TransformerMixin):

"""Base class for all spline basis expansions."""

def __init__(self, max=None, min=None, n_knots=None, n_params=None, knots=None):

if knots is None:

if not n_knots:

n_knots = self._compute_n_knots(n_params)

knots = np.linspace(min, max, num=(n_knots + 2))[1:-1]

max, min = np.max(knots), np.min(knots)

self.knots = np.asarray(knots)

@property

def n_knots(self):

return len(self.knots)

def fit(self, *args, **kwargs):

return self

class NaturalCubicSpline(AbstractSpline):

"""Apply a natural cubic basis expansion to an array.

The features created with this basis expansion can be used to fit a

piecewise cubic function under the constraint that the fitted curve is

linear *outside* the range of the knots.. The fitted curve is continuously

differentiable to the second order at all of the knots.

This transformer can be created in two ways:

- By specifying the maximum, minimum, and number of knots.

- By specifying the cutpoints directly.

If the knots are not directly specified, the resulting knots are equally

space within the *interior* of (max, min). That is, the endpoints are

*not* included as knots.

Parameters

----------

min: float

Minimum of interval containing the knots.

max: float

Maximum of the interval containing the knots.

n_knots: positive integer

The number of knots to create.

knots: array or list of floats

The knots.

"""

def _compute_n_knots(self, n_params):

return n_params

@property

def n_params(self):

return self.n_knots - 1

def transform(self, X, **transform_params):

X_spl = self._transform_array(X)

if isinstance(X, pd.Series):

col_names = self._make_names(X)

X_spl = pd.DataFrame(X_spl, columns=col_names, index=X.index)

return X_spl

def _make_names(self, X):

first_name = "{}_spline_linear".format(X.name)

rest_names = ["{}_spline_{}".format(X.name, idx)

for idx in range(self.n_knots - 2)]

return [first_name] + rest_names

def _transform_array(self, X, **transform_params):

X = X.squeeze()

try:

X_spl = np.zeros((X.shape[0], self.n_knots - 1))

except IndexError: # For arrays with only one element

X_spl = np.zeros((1, self.n_knots - 1))

X_spl[:, 0] = X.squeeze()

def d(knot_idx, x):

def ppart(t): return np.maximum(0, t)

def cube(t): return t*t*t

numerator = (cube(ppart(x - self.knots[knot_idx]))

- cube(ppart(x - self.knots[self.n_knots - 1])))

denominator = self.knots[self.n_knots - 1] - self.knots[knot_idx]

return numerator / denominator

for i in range(0, self.n_knots - 2):

X_spl[:, i+1] = (d(i, X) - d(self.n_knots - 2, X)).squeeze()

return X_spl

def Knot(x,y,knots=15,plot=1): # x and y are numpy arrays of equal length, knots is a variable controlling smoothing degree, plot is a bool controlling graph production

from matplotlib import pyplot as plt

import numpy as np

import Supplementary_Script_1 as KF

# The number of knots can be used to control the amount of smoothness

model_15 = KF.get_natural_cubic_spline_model(x, y, minval=min(x), maxval=max(x), n_knots=knots)

y_est_15 = model_15.predict(x)

if plot==1:

plt.plot(x, y, ls='', marker='.', label='originals')

plt.plot(x, y_est_15, marker='.', label=('n_knots = '+str(knots)))

plt.legend(); plt.show()

return model_15 # function returns the modelled curve, which can then make predictions using the predict method

**Supplementary Equations 2**

from matplotlib import pyplot as plt

import numpy as np

import Supplementary_Script_1 as KF

import csv

ThresholdProp=0.25 # Minimum proportion of juvenile/female siskins required for date storage by alternative date scoring method.

ListOfProportions=[] # Proportion of juvenile/female siskins for each entry

ListOfDaysOfYear=[] # Day of year for each entry

ListOfYears=[] # Year for each entry

AllYears=set([]) # List of every year represented in the data

KnotColumn=4 # Column index (starting at 0) of the file that is to be knotted

with open('C:/Users/User/Desktop/Sessions.csv', newline='') as csvfile: # csvfile must be sorted in ascending order on the x axis (i.e. by day of year)

siskins = csv.reader(csvfile, delimiter=' ', quotechar='|')

header=1

for row in siskins:

if header==1:

header=0

else:

Qrow=row[0]

values=Qrow.split(",")

ListOfProportions.append(float(values[KnotColumn]))

ListOfDaysOfYear.append(int(values[1]))

ListOfYears.append(int(values[0]))

if int(values[0]) not in AllYears:

AllYears.add(int(values[0]))

x=np.asarray(ListOfDaysOfYear)

y=np.asarray(ListOfProportions)

KnotModel=KF.Knot(x,y,knots=8)

ModdingY=0 # bool. Controls whether graph is allowed to be stretched in y (1) or not (0).

for year in AllYears:

PosWYear=[] # List of positions in the previous lists where the year is the year of interest

PropInYear=[] # List of proportions of juvenile/female siskins in each catch in the year of interest

DayInYear=[] # List of each catch date in the year of interest

index=0

for yearRun in ListOfYears:

if yearRun==year:

PosWYear.append(index)

else:

pass

index=index+1

for indexed in PosWYear:

PropInYear.append(ListOfProportions[indexed])

DayInYear.append(ListOfDaysOfYear[indexed])

indexID=-1 # Block determines the first instance of a ringing session in the list, in each year, where the proportion of juvenile/female siskins exceeded a threshold, ThresholdProp

KeyIndex=-1

for Prop in PropInYear:

indexID=indexID+1

if Prop>ThresholdProp and KeyIndex==-1: # The sign (< or >) may need modified depending on the question being asked

KeyIndex=indexID

if KeyIndex==-1:

ThresholdDate="No Sufficient Catches this Year."

else:

ThresholdDate=DayInYear[KeyIndex]

xBase=np.asarray(DayInYear)

yBase=np.asarray(PropInYear)

OptCycles=0

OptCyclesMax=3 # Number of cycles in which misfit is minimised, with the window shrinking each time.

YearModCentre=0 # Variable that tracks the optimum additive modifier in x

AmpModCentre=2 # Variable that tracks the optimum multiplicative modifier in y

while OptCycles<OptCyclesMax:

MinError=100000000000000000000000000000000000000000 # A very high number, designed never to be exceeded in the analysis.

YearMod=np.linspace(YearModCentre-(100/(10**OptCycles)),YearModCentre+(100/(10**OptCycles)),402) # Array of possible x-axis corrections: additive

if ModdingY==1:

if OptCycles==0:

AmpMod=np.linspace(AmpModCentre-(2/(10**OptCycles)),AmpModCentre+(2/(10**OptCycles)),220) # Array of possible y-axis corrections: multiplicative

else:

AmpMod=np.linspace(AmpModCentre-(5/(10**OptCycles)),AmpModCentre+(5/(10**OptCycles)),220) # Multiplicative range is relatively larger after the first cycle

else:

AmpMod=[1,1]

for xMod in YearMod: # For every additive modifier to be checked

for yMod in AmpMod: # For every multiplicative modifier to be checked

x=xBase+xMod

Predicted=yMod*(KnotModel.predict(x)) # Make predictions using those modifiers

CumError=0

PredictionIndex=-1

for prediction in Predicted: # Evaluate the cumulative error of those predictions

PredictionIndex=PredictionIndex+1

CumError=CumError+(prediction-yBase[PredictionIndex])**2

if CumError<MinError: # Store the values with the lowest cumulative error

MinError=CumError

YearModCentre=xMod

AmpModCentre=yMod

OptCycles=OptCycles+1

print(year)

print(YearModCentre) # Positive indicates an early year

print(AmpModCentre)

print(ThresholdDate)

**Supplementary Equations 3**

with open("C:/Users/User/Desktop/Ringing_Records.csv") as infile: # Adjust to the path to the input ringing file

with open("C:/Users/User/Desktop/Session_Log.txt","w+") as outfile: # Adjust to the path for the output

outfile.writelines(["Year, Day of Year, n(juvenile siskins), n(all siskins), p(juvenile siskins)\n"]) # Adjust if the sampling conditions are different

header=1

CurJuvSiskins=0

CurBirds=1

CurYear=0

CurMonth=0

CurDay=0

MinBirds=15 # Minimum number of captured birds for a session record to be made

for line in infile:

CorruptEntry=0

if header==1:

header=0

else:

try:

splitline=line.split(",") # The values in the following splitline functions can be adjusted as required for the input file.

year=int(splitline[7])

month=int(splitline[6])

day=int(splitline[5])

species=splitline[2]

euring=int(splitline[3])

except:

CorruptEntry=1

print("Corrupted Entry, Skipping:")

print(line)

if CorruptEntry==0:

if year!=CurYear or month!=CurMonth or day!=CurDay:

if CurBirds>=MinBirds:

DayOfYear=CurDay

if CurMonth>1:

DayOfYear=DayOfYear+31

if CurMonth>2:

DayOfYear=DayOfYear+28

if year%4==0:

DayOfYear=DayOfYear+1

if CurMonth>3:

DayOfYear=DayOfYear+31

if CurMonth>4:

DayOfYear=DayOfYear+30

if CurMonth>5:

DayOfYear=DayOfYear+31

if CurMonth>6:

DayOfYear=DayOfYear+30

if CurMonth>7:

DayOfYear=DayOfYear+31

if CurMonth>8:

DayOfYear=DayOfYear+31

if CurMonth>9:

DayOfYear=DayOfYear+30

if CurMonth>10:

DayOfYear=DayOfYear+31

if CurMonth>11:

DayOfYear=DayOfYear+30

if CurBirds>0:

PropJuvSis=CurJuvSiskins/CurBirds

outfile.writelines([str(CurYear)+","+str(DayOfYear)+","+str(CurJuvSiskins)+","+str(CurBirds)+","+str(PropJuvSis)+"\n"])

else:

outfile.writelines([str(CurYear)+","+str(DayOfYear)+","+str(CurJuvSiskins)+","+str(CurBirds)+",n/a\n"])

CurYear=year

CurMonth=month

CurDay=day

CurBirds=0

CurJuvSiskins=0

else:

pass

if species=="Siskin" and euring>2: # Adjust this to adjust the script's count of the denominator birds

CurBirds=CurBirds+1

if species=="Siskin" and euring==3: # Adjust this to adjust the script's count of the numerator birds

CurJuvSiskins=CurJuvSiskins+1

**Supplementary Equations 4**

from matplotlib import pyplot as plt

import numpy as np

import Knotter_Functions as KF

import csv

ThresholdProp=0.25 # Minimum proportion of female siskins required for date storage by alternative date scoring method.

ListOfProportions=[] # Proportion of female siskins for each entry

ListOfDaysOfYear=[] # Day of year for each entry

ListOfYears=[] # Year for each entry

AllYears=set([]) # List of every year represented in the data

KnotColumn=3 # Column index (starting at 0) of the file that is to be knotted

with open('C:/Users/User/Session_Log.csv', newline='') as csvfile: # csvfile must be sorted in ascending order on the x axis

siskins = csv.reader(csvfile, delimiter=' ', quotechar='|')

header=1

for row in siskins:

if header==1:

header=0

else:

Qrow=row[0]

values=Qrow.split(",")

ListOfProportions.append(float(values[KnotColumn]))

ListOfDaysOfYear.append(int(values[1]))

ListOfYears.append(int(values[0]))

if int(values[0]) not in AllYears:

AllYears.add(int(values[0]))

x=np.asarray(ListOfDaysOfYear)

y=np.asarray(ListOfProportions)

KnotModel=KF.Knot(x,y,knots=8)

ModdingY=0 # bool. Controls whether graph is allowed to be stretched in y (1) or not (0).

for year in AllYears:

PosWYear=[] # List of positions in the previous lists where the year is the year of interest

PropInYear=[] # List of proportions of female siskins in each catch in the year of interest

DayInYear=[] # List of each catch date in the year of interest

index=0

for yizzle in ListOfYears:

if yizzle==year:

PosWYear.append(index)

else:

pass

index=index+1

for indexed in PosWYear:

PropInYear.append(ListOfProportions[indexed])

DayInYear.append(ListOfDaysOfYear[indexed])

indexID=-1 # Block determines the first instance of a ringing session in the list, in each year, where the proportion of female siskins falls below a threshold, ThresholdProp

KeyIndex=-1

for Prop in PropInYear:

indexID=indexID+1

if Prop<ThresholdProp and KeyIndex==-1: # The sign (< or >) may need modified depending on the question being asked

KeyIndex=indexID

if KeyIndex==-1:

ThresholdDate="No Sufficient Catches this Year."

else:

ThresholdDate=DayInYear[KeyIndex]

xBase=np.asarray(DayInYear)

yBase=np.asarray(PropInYear)

OptCycles=0

OptCyclesMax=3 # Number of cycles in which misfit is minimised, with the window shrinking each time.

YearModCentre=0 # Variable that tracks the optimum additive modifier in x

AmpModCentre=2 # Variable that tracks the optimum multiplicative modifier in y

while OptCycles<OptCyclesMax:

MinError=100000000000000000000000000000000000000000

YearMod=np.linspace(YearModCentre-(100/(10**OptCycles)),YearModCentre+(100/(10**OptCycles)),402) # Array of possible x-axis corrections: additive

if ModdingY==1:

if OptCycles==0:

AmpMod=np.linspace(AmpModCentre-(2/(10**OptCycles)),AmpModCentre+(2/(10**OptCycles)),220) # Array of possible y-axis corrections: multiplicative

else:

AmpMod=np.linspace(AmpModCentre-(5/(10**OptCycles)),AmpModCentre+(5/(10**OptCycles)),220) # Multiplicative range is relatively larger after the first cycle

else:

AmpMod=[1,1]

for xMod in YearMod: # For every additive modifier to be checked

for yMod in AmpMod: # For every multiplicative modifier to be checked

x=xBase+xMod

Predicted=yMod*(KnotModel.predict(x)) # Make predictions using those modifiers

CumError=0

PredictionIndex=-1

for prediction in Predicted: # Evaluate the cumulative error of those predictions

PredictionIndex=PredictionIndex+1

CumError=CumError+(prediction-yBase[PredictionIndex])**2

if CumError<MinError: # Store the values with the lowest cumulative error

MinError=CumError

YearModCentre=xMod

AmpModCentre=yMod

OptCycles=OptCycles+1

print(year)

print(YearModCentre) # Positive indicates an early year

print(AmpModCentre)

print(ThresholdDate)

**Supplementary Equations 5**

with open("C:/Users/User/Ringing_Records.csv") as infile:

with open("C:/Users/User/Session_Log.txt","w+") as outfile:

outfile.writelines(["Year, n(5 siskins), n(6 siskins), 5s/5s+6s\n"])

header=1

CurJuvSiskins=0

CurBirds=1

CurYear=0

CurMonth=0

MinBirds=0 # Minimum number of captured birds for a record to be made

for line in infile:

CorruptEntry=0

if header==1:

header=0

else:

try:

splitline=line.split(",")

year=int(splitline[7])

month=int(splitline[6])

day=int(splitline[5])

species=splitline[2]

euring=int(splitline[3])

except:

CorruptEntry=1

print("Corrupted Entry, Skipping:")

print(line)

if CorruptEntry==0:

if year!=CurYear:

if CurBirds+CurJuvSiskins>MinBirds:

PropJuvSis=CurJuvSiskins/(CurBirds+CurJuvSiskins)

outfile.writelines([str(CurYear)+","+str(CurJuvSiskins)+","+str(CurBirds)+","+str(PropJuvSis)+"\n"])

CurYear=year

CurMonth=month

CurBirds=0

CurJuvSiskins=0

else:

pass

if month>=3:

if species=="Siskin" and euring==6:

CurBirds=CurBirds+1

if species=="Siskin" and euring==5:

CurJuvSiskins=CurJuvSiskins+1

if CurBirds+CurJuvSiskins>MinBirds:

PropJuvSis=CurJuvSiskins/(CurBirds+CurJuvSiskins)

outfile.writelines([str(CurYear)+","+str(CurJuvSiskins)+","+str(CurBirds)+","+str(PropJuvSis)+"\n"])

**Supplementary Table 1. Weights and wing lengths of juvenile siskins in different months of capture**

| Month | Number measured | Mean weight (g) | S.D. | 95% confidence interval | Wing length (mm) | Standard deviation | 95% confidence interval |
| --- | --- | --- | --- | --- | --- | --- | --- |
| April | 6 | 11.94 | 0.302 | 11.70-12.18 | 71.83 | 0.983 | 71.04-72.62 |
| May | 525 | 11.75 | 0.656 | 11.69-11.81 | 71.62 | 1.717 | 71.47-71.77 |
| June | 1698 | 11.96 | 0.755 | 11.92-12.00 | 71.89 | 1.719 | 71.81-71.97 |
| July | 1071 | 12.26 | 0.760 | 12.21-12.31 | 72.07 | 1.754 | 71.96-72.18 |
| August | 351 | 12.60 | 0.822 | 12.51-12.69 | 71.93 | 1.781 | 71.74-72.12 |
| September | 171 | 12.67 | 0.883 | 12.54-12.80 | 71.67 | 1.782 | 71.40-71.94 |


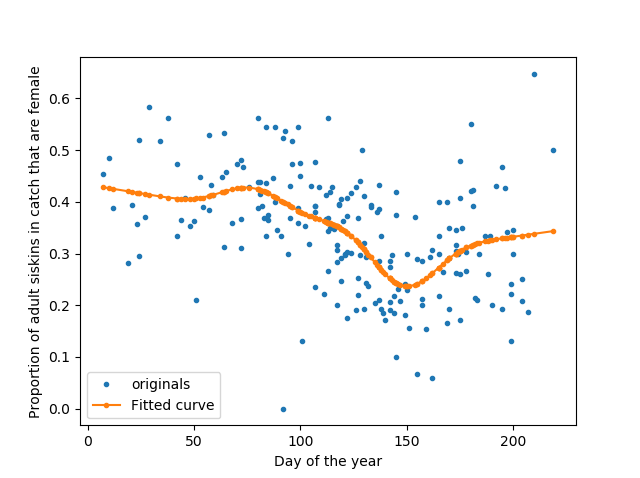


**Supplementary Figure 1. Adult sex ratio throughout the year.** Proportion of all captured and sexed adult siskins identified as female in catches with at least 11 adults at different times of year, from 2005 to 2020. Curve fitted to the data using misfit minimization.


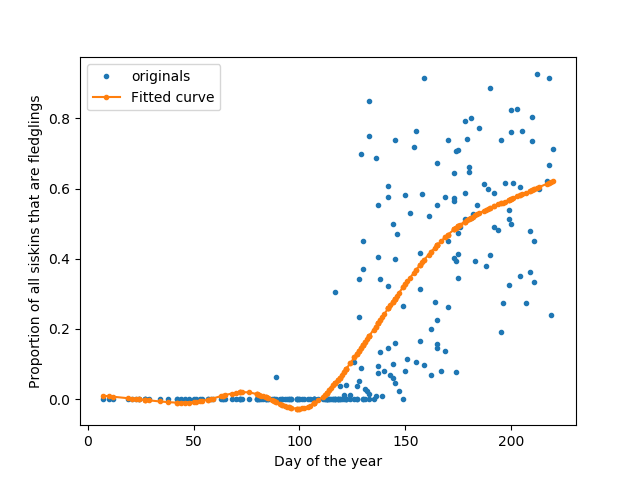


**Supplementary Figure 2. Fledgling abundance throughout the year.** Proportion of all captured siskins identified as fledglings on each day of the year. Captures after day 260 were excluded because siskins were rare in catches after that date. Curve fitted to the data. Data run from 2005-2020.


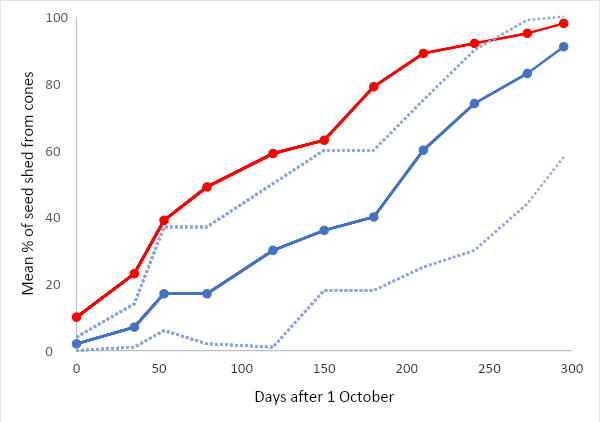


**Supplementary Figure 3. Mean proportion of seed shed by Sitka spruce cones in 2006-07.** West Lamberkine Wood, Perthshire in red, Argyll Forest Park, Tarbet, Argyll & Bute in blue, with maximum and minimum values for Tarbet.


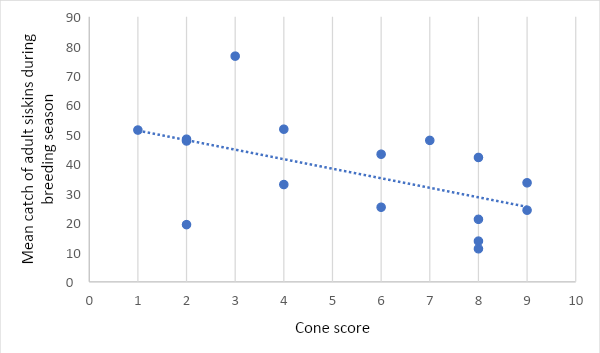


**Supplementary Figure 4. Cone crop impact on adult catch.** Relationship between the mean catch of adult siskins during the breeding season and Sitka spruce cone score. R^2^=0.2778.
